# Inhibition of chymotrypsin-like activity of the proteasome by ixazomib prevents mitochondrial dysfunction during myocardial ischemia

**DOI:** 10.1371/journal.pone.0233591

**Published:** 2020-05-26

**Authors:** Gina Sánchez, Stefanie Chalmers, Xavier Ahumada, Luis Montecinos, Ivonne Olmedo, Veronica Eisner, Ana Riveros, Marcelo J. Kogan, Sergio Lavandero, Zully Pedrozo, Paulina Donoso

**Affiliations:** 1 Programa de Fisiopatología, Instituto de Ciencias Biomédicas, Facultad de Medicina, Universidad de Chile, Santiago, Chile; 2 Centro de Estudios en Ejercicio, Metabolismo y Cáncer, Facultad de Medicina, Universidad de Chile, Santiago, Chile; 3 Programa de Fisiología y Biofísica, Instituto de Ciencias Biomédicas, Facultad de Medicina, Universidad de Chile, Santiago, Chile; 4 Departamento de Biología Celular y Molecular, Facultad de Ciencias Biológicas, Pontificia Universidad Católica de Chile, Santiago, Chile; 5 Departamento de Química Farmacológica y Toxicológica, Facultad Ciencias Químicas y Farmacéuticas Universidad de Chile, Santiago, Chile; 6 Departamento de Bioquímica y Biología Molecular, Facultad Ciencias Químicas y Farmacéuticas, Universidad de Chile, Santiago, Chile; 7 Advanced Center for Chronic Diseases (ACCDiS), Santiago, Chile; Virginia Commonwealth University, UNITED STATES

## Abstract

The heart is critically dependent on mitochondrial respiration for energy supply. Ischemia decreases oxygen availability, with catastrophic consequences for cellular energy systems. After a few minutes of ischemia, the mitochondrial respiratory chain halts, ATP levels drop and ion gradients across cell membranes collapse. Activation of cellular proteases and generation of reactive oxygen species by mitochondria during ischemia alter mitochondrial membrane permeability, causing mitochondrial swelling and fragmentation and eventually cell death. The mitochondria, therefore, are important targets of cardioprotection against ischemic injury. We have previously shown that ixazomib (IXA), a proteasome inhibitor used for treating multiple myeloma, effectively reduced the size of the infarct produced by global ischemia in isolated rat hearts and prevented degradation of the sarcoplasmic reticulum calcium release channel RyR2. The aim of this work was to further characterize the protective effect of IXA by determining its effect on mitochondrial morphology and function after ischemia. We also quantified the effect of IXA on levels of mitofusin-2, a protein involved in maintaining mitochondrial morphology and mitochondria-SR communication. We found that mitochondria were significantly preserved and functional parameters such as oxygen consumption, the ability to generate a membrane potential, and glutathione content were improved in mitochondria isolated from hearts perfused with IXA prior to ischemia. IXA also blocked the release of cytochrome c observed in ischemia and significantly preserved mitofusin-2 integrity. These beneficial effects resulted in a significant decrease in the left ventricular end diastolic pressure upon reperfusion and a smaller infarct in isolated hearts.

## Introduction

The search for protective measures against cardiac ischemia/reperfusion injury has been a matter of active research for the last 30 years. Therapeutic interventions at the onset of reperfusion can limit the damage produced by ischemia, but outcomes after reperfusion remain critically dependent on the degree and duration of ischemia [1]. Cardiac tissue is highly dependent on mitochondrial oxidative phosphorylation for energy production, and when oxygen availability is low, the mitochondrial respiratory rate falls, ATP levels drop, and whole-cell homeostasis is impaired. Alteration of ionic gradients across mitochondrial membranes causes loss of membrane potential, swelling and disorganization of cristae, fragmentation of mitochondria and the release of molecules that eventually produce cell death [2,3]. Therefore, therapeutic measures to prevent or delay mitochondrial damage during ischemia would increase the resistance of the heart to ischemic injury and would undoubtedly be an advantage in those cases where myocardial ischemia can be programmed in advance, such as heart surgeries or organ transplantation.

Many proteins are degraded during ischemia by the proteolytic action of the 20S proteasome, including ryanodine receptors (RyR2), the calcium release channels located in the sarcoplasmic reticulum (SR). RyR2 are rapidly oxidized and degraded during myocardial ischemia, significantly impacting cardiac performance [4,5]. The 20S proteasome has three main proteolytic activities: chymotrypsin-like (CT-like), caspase-like and trypsin-like activities. In a recent work, we showed that after 30 minutes of global ischemia in isolated rat hearts, CT-like activity increases by 60%, while caspase-like and trypsin-like catalytic activities remain unchanged [6]. Inhibition of CT-like activity with ixazomib (IXA), a proteasome inhibitor currently used in patients with multiple myeloma [7], prevents RyR2 degradation during ischemia and significantly improves cell survival after ischemia/reperfusion [6]. In the heart, the SR and mitochondria are physically connected, forming microdomains that allow for the transfer of calcium from the SR to the mitochondria so that mitochondrial energy production can satisfy energy requirements [8,9]. Several proteins are involved in the organization of SR-mitochondria microdomains, including RyR2 and mitofusin-2 (Mfn2), a GTPase localized to the microdomains known as mitochondrial associated membranes, that include the SR [10,11]. Mfn2 also regulates mitochondrial fusion [12] and respiratory chain function by maintaining mitochondrial levels of coenzyme Q [13]. Mitochondrial function is therefore critically dependent on Mfn2 integrity. Under stress conditions, such as ischemia, Mfn2 is phosphorylated and degraded by the proteasome [14]. As a consequence of the degradation of this and other proteins, mitochondria undergo fragmentation and degradation. The effect of IXA on post-ischemia mitochondrial function has not been investigated before and since this inhibitor effectively protected RyR2, one of the proteins involved in the SR-mitochondrial association, we aimed to determine whether Mfn2 degradation can be prevented by IXA; and to evaluate the effect of the drug on the functional and morphologic changes to the mitochondria that occur during ischemia but before reperfusion in isolated rat hearts.

## Materials and methods

Male Sprague-Dawley (SD) rats (250–300 g) were obtained from the animal facility of the University of Chile, School of Medicine. All study procedures were approved by the Institutional Ethics Committee of the School of Medicine (Protocol CBA #541 FMUCH) and performed according to the Guide for the Care and Use of Laboratory Animals published by the U.S. National Institutes of Health (NIH, 2011).

### Experimental protocol

Hearts were excised under deep anesthesia with pentobarbital (80 mg/kg, intraperitoneal) and rapidly perfused at 37°C via the ascending aorta at a constant flow of 10–14 mL/minute, using a peristaltic infusion pump with Krebs-Henseleit solution containing (in mmol/L): 128.3 NaCl, 4.7 KCl, 1.35 CaCl_2_, 1.1 MgSO_4_, 20.2 NaHCO_3_, 0.4 NaH_2_PO_4_, and 11.1 glucose, at a pH of 7.4, equilibrated with a gas mixture of 95% O_2_/5% CO_2_. A latex balloon was inserted into the left ventricle and connected to a pressure transducer to measure hemodynamic parameters.

Experimental groups. Control group (N = 15): Hearts were perfused with Krebs buffer solution for 50 minutes. Ischemia group (N = 15): hearts were stabilized for 20 minutes with Krebs buffer solution, followed by 30 minutes of global ischemia at 37°C. Control group with IXA (N = 9): Hearts were stabilized for 10 minutes with Krebs buffer solution, followed by 10 minutes of perfusion with Krebs plus the proteasome inhibitor ixazomib (IXA, 0.10 μmol/L), and then 30 minutes of perfusion with Krebs buffer. Ischemia group with IXA (N = 9): Hearts were stabilized for 10 minutes with Krebs buffer solution, followed by 10 minutes of perfusion with IXA, and then 30 minutes of global ischemia.

Immediately after ischemia, or the equivalent time in controls, heart ventricles were snap-frozen in liquid nitrogen, reduced to powder under liquid N_2_, and kept under argon atmosphere at -80°C.

### Infarct size measurement

Hearts subjected to ischemia in the absence (N = 10) or in the presence of IXA (N = 10) were reperfused with Krebs solution for 60 minutes and then perfused with triphenyltetrazolium chloride (TTC, Sigma-Aldrich, St. Louis, MO) to measure the infarct size as described previously [15].

### Proteasome activity

Whole-ventricle homogenates were prepared from frozen tissue, and chymotrypsin-like activity of the proteasome was determined as described previously [6,16].

### Quantification of mitochondrial DNA

Total DNA from ventricular tissue was obtained using DNAzol® (Invitrogen, Carlsbad, CA, USA) following the manufacturer’s instructions. The homogenate was centrifuged at 10,000 x g for 10 minutes at 4ºC. Ethanol was added to the supernatant to precipitate total DNA. Mitochondrial DNA (mtDNA) was quantified by detecting D-loop with the PCR primers, F: 5’GGT TCT TAC TTC AGG GCC ATC A-3’ and R: 5’-GAT TAG ACC CGT TAC CAT CGA GAT-3’. β-actin, used as a housekeeping gene, was detected with specific primers: F: 5’-GGG ATG TTT GCT CCA ACC AA-3’ and R: 5’-GCG CTT TTG ACT CAA GGA TTT AA-3’. The 2-ΔΔCt method was used to calculate relative transcript abundance.

### Preparation of mitochondria

Frozen ventricles were homogenized with a Dounce glass Teflon homogenizer with 6 volumes of buffer solution consisting of (in mmol/L): 225 mannitol, 75 sucrose, 0.5 EGTA-K, 30 TRIS-HCl, at a pH 7.4, with 0.5% BSA. The crude mitochondrial fraction was isolated by differential centrifugation according the procedure described by Wieckowski [17]. The supernatant of the mitochondrial fraction was saved to measure cytochrome c content by Western blot.

### Oxygen consumption

Mitochondrial fractions were incubated in a buffer containing (in mmol/L): 225 mannitol, 75 sucrose, 0.5 EGTA-K, 30 TRIS-HCl, at pH 7.4, with 0.5% BSA supplemented with 25 mmol/L succinate and 1.65 mmol/L ADP and the fluorescent probe MitoXpress a 37ºC. Samples were covered with a layer of mineral oil, according to the manufacturer's recommendations (MitoXpress ®, catalog # 600800, Cayman Chemical, MI-USA) and the light excited at 380 nm and emitted at 650 nm was measured in a plate reader. The results were normalized with respect to controls and expressed in arbitrary units of fluorescence/minute/mg of protein.

### Mitochondrial membrane potential

Mitochondrial fractions were incubated in a solution containing 110 mM KCl, 10 mM ATP, 10 mM MgCl_2_, 10 mM sodium succinate, 1 mM EGTA and 20 mM MOPS, pH 7.5 and the fluorescent probe JC-1 (Catalog Number J4519, Sigma-Aldrich, St. Louis, MO) according manufacturer´s recommendations. JC-1 aggregates in polarized mitochondria and changes its fluorescent emission. Fluorescence was excited at 490 and the ratio of emitted light at 590/540 nm was measured. The results were expressed in arbitrary units of fluorescence/mg protein.

### Glutathione content

Total glutathione (GSH + GSSG) concentration was determined in isolated mitochondria as described by Griffith [18].

### Western blot analysis

Proteins were separated by electrophoresis in polyacrylamide gel (8 or 15% gels), transferred to PVDF membranes (Bio-Rad), and immunoblotted. The primary antibodies used were: anti-Mfn2 (ab50483, lot# GR297107-2, Abcam, Cambridge, UK), anti-cytochrome c (C5723, lot# 038K1427, Sigma Chemicals Co., St. Louis, MO), anti-GAPDH (G9547, lot# 026M4836V, Sigma Chemicals Co., St. Louis, MO). Antigen-antibody reactions were detected by ECL (Amersham Biosciences), and blots were quantified using Image Lab software. Results were normalized to GADPH. Protein concentration was determined using the bicinchoninic acid assay (Pierce BCA Protein Assay Kit; Thermo Scientific, Rockford, IL).

### Transmission Electron Microscope (TEM)

Immediately after the experiment, the ischemic hearts were perfused for 30 minutes with a solution of 3% glutaraldehyde in 0.1 mol/L sodium phosphate buffer, at a pH of 7.3, and treated with 1% osmium tetroxide. Samples were dehydrated in ethanol solutions of increasing concentrations and finally placed in EPON resin. Sections, 60-nm thick, were cut from the tissue and mounted on electron microscopy grids. Images were acquired using a transmission electron microscope at 80 kV (Philips Tecnai 12, at the Advanced Microscopy Facility, Pontificia Universidad Católica) or in a high-resolution scanning electron microscope (Inspect F50, FEI, at the Microscopy Facility of the Facultad de Ciencias Químicas, Universidad de Chile) at 10kV with a STEM detector.

### Statistical analysis

Data are expressed as mean ± SEM. Data were analyzed using Kruskal-Wallis test followed by Dunn´s test. When comparing two groups Student’s t-test was used. Differences were considered significant at p<0.05.

## Results

### Inhibition of CT-like activity of the proteasome prevents mitofusin-2 degradation during ischemia

**Fig 1:**
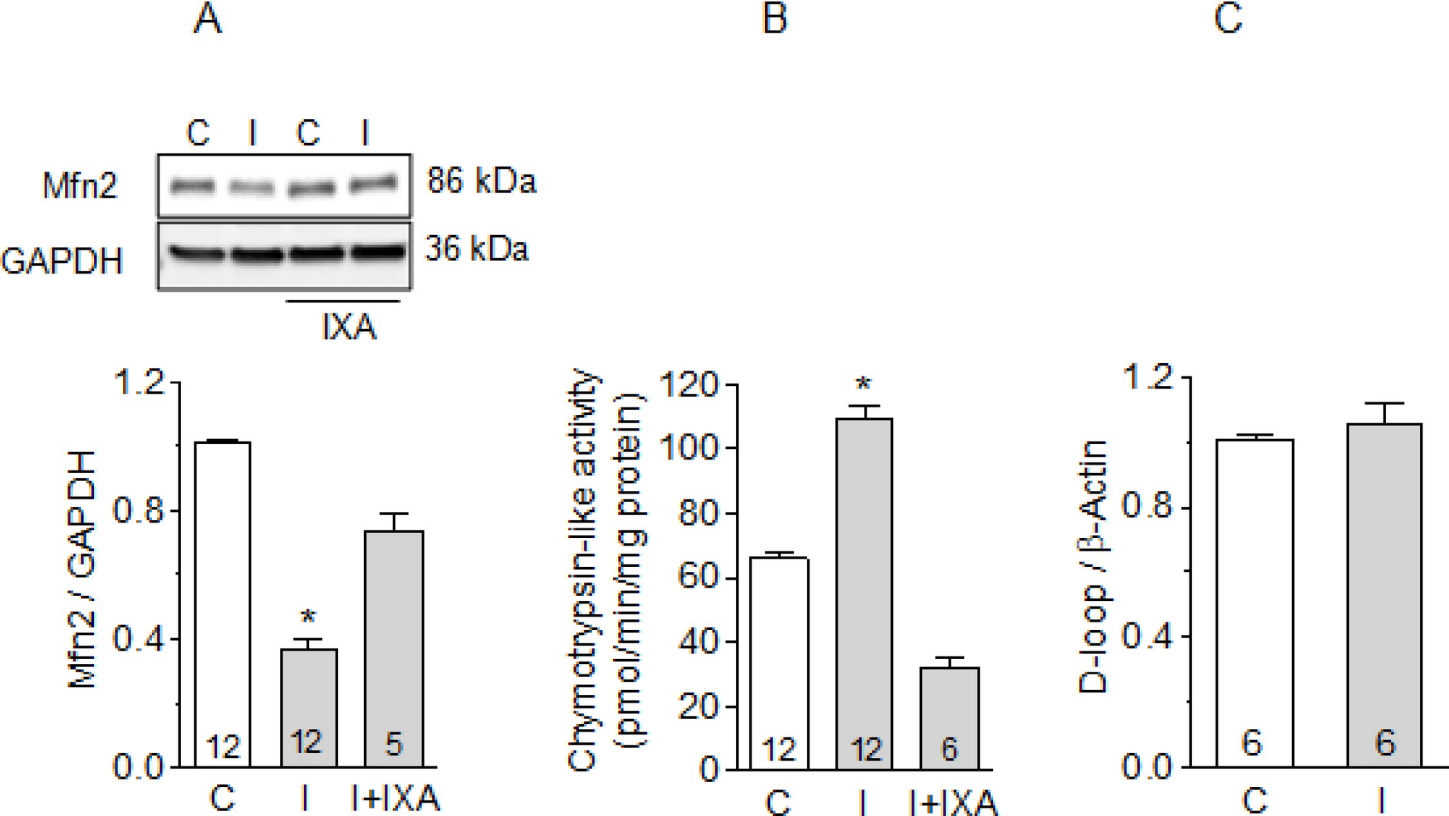
Effect of ixazomib on mitofusin-2 content and proteasome activity after ischemia. (A) Mitofusin-2 content in control hearts, after 30 minutes of ischemia, or after ischemia in hearts previously perfused with IXA. Representative Western blots are shown at the top. (B) Chymotrypsin-like activity of the proteasome in control hearts, after ischemia, or after ischemia in hearts previously perfused with IXA. (C) mtDNA (D-loop) to nDNA (β-actin) by real-time quantitative PCR in control or ischemic hearts. Labels: C, control hearts, I: ischemic hearts. Bars show the average ± SEM of the number of hearts shown in each bar. *p<0.05 vs. all other conditions. #p<0.05 vs. C; Kruskal-Wallis test followed by Dunn`s test.

We found that Mfn2 was highly sensitive to ischemia, decreasing by 60% after 30 minutes of global ischemia in isolated hearts. This decrease was prevented when the hearts were previously perfused with IXA (Fig 1A). As shown previously, the increase in proteasomal CT-like activity observed after ischemia was also inhibited by IXA (Fig 1B), suggesting that Mfn2 was degraded by the proteasome. This effect is similar to the previously-reported inhibition of RyR2 degradation by IXA during ischemia [6]. We did not observe changes in mitochondrial DNA after 30 minutes of ischemia (Fig 1C), suggesting that mitochondrial mass did not change during this period. Likewise, we found no degradation of other mitochondrial proteins such as Mitofusin-1 or Fis-1, both localized to the outer mitochondrial membrane, nor in OPA-1 or COX IV, both integral proteins of the inner mitochondrial membrane or in Cyclofilin D, a mitochondrial matrix protein, suggesting that the degradation of Mfn2 is not the result of the generalized degradation of proteins after ischemia (S1 Fig).

### Ixazomib preserves mitochondrial function during ischemia

**Fig 2:**
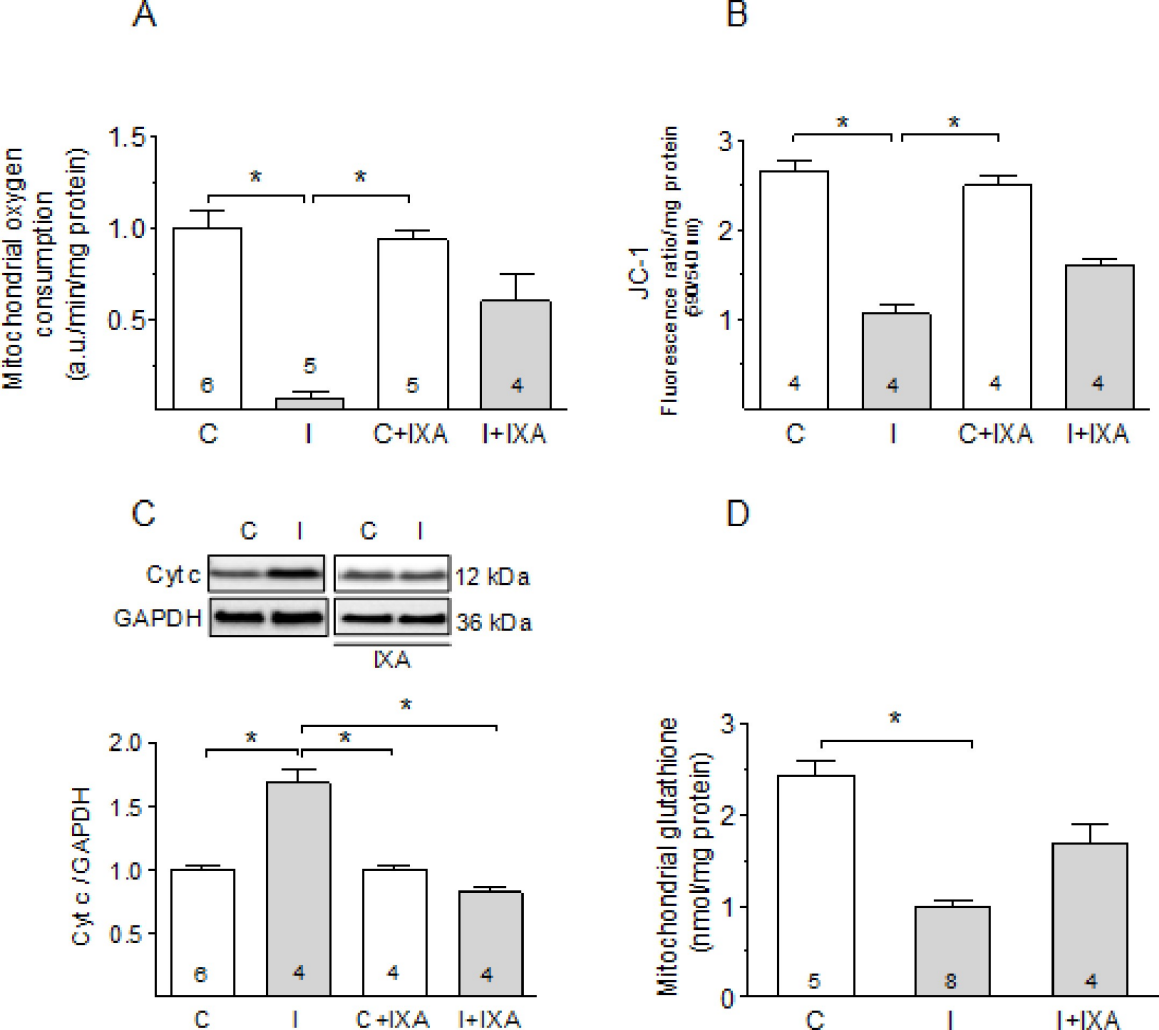
Mitochondrial function after ischemia is preserved by ixazomib. (A) Oxygen consumption, (B) JC-1 fluorescence ratio 590/540, (C) cytosolic cyt c and (D) mitochondrial glutathione content in mitochondria isolated from control hearts or after 30 minutes of ischemia with or without previous perfusion with IXA. Labels C: control, I: ischemia. Oxygen consumption and Cyt c were expressed relative to controls. Number of measurements in different hearts is shown in each bar. *p<0.05. Kruskal-Wallis test followed by Dunn`s test.

To evaluate the effect of IXA on the functional status of the mitochondria, we measured oxygen consumption and the capacity of isolated mitochondria to generate a membrane potential in the presence of respiratory substrates and cofactors. Oxygen consumption by mitochondria isolated from ischemic hearts was reduced to less than 10% of control levels but showed only a 40% reduction when ischemia was performed in the presence of IXA (Fig 2A). To evaluate the ability to generate a potential difference across the inner mitochondrial membrane we measured the accumulation of the lipophilic cationic dye JC-1. Mitochondria isolated from ischemic hearts accumulated significantly less JC-1 suggesting that they produced a smaller membrane potential difference than controls (Fig 2B). IXA significantly prevented the decrease in JC-1 accumulation observed after ischemia (Fig 2B). We also measured cytochrome c (cyt c) levels in the supernatant of the mitochondrial fraction as another measure of mitochondrial dysfunction. We observed a significant increase in cyt c content in the soluble fraction of ischemic hearts (Fig 2C). This effect was completely prevented by IXA, confirming the protective effect of the inhibitor on the mitochondria.

An important cause of ischemia-induced mitochondrial dysfunction is the generation of ROS, which oxidize lipids and proteins. Mitochondria maintain their own pool of glutathione [19], which is involved in the protection of thiol groups, detoxification of peroxides, and other redox reactions in the mitochondrial matrix. Mitochondrial glutathione content is an indirect index of ROS generation. We found that the glutathione (GSH + GSSG) content in isolated mitochondria was greatly decreased after ischemia and this was largely prevented by IXA (Fig 2D). IXA did not modify any of the above-mentioned mitochondrial parameters when infused into control hearts. Taken together, these results suggest that IXA protects mitochondria from the damage produced by ischemia.

### Ixazomib attenuates the morphological changes to the mitochondria produced by ischemia

**Fig 3:**
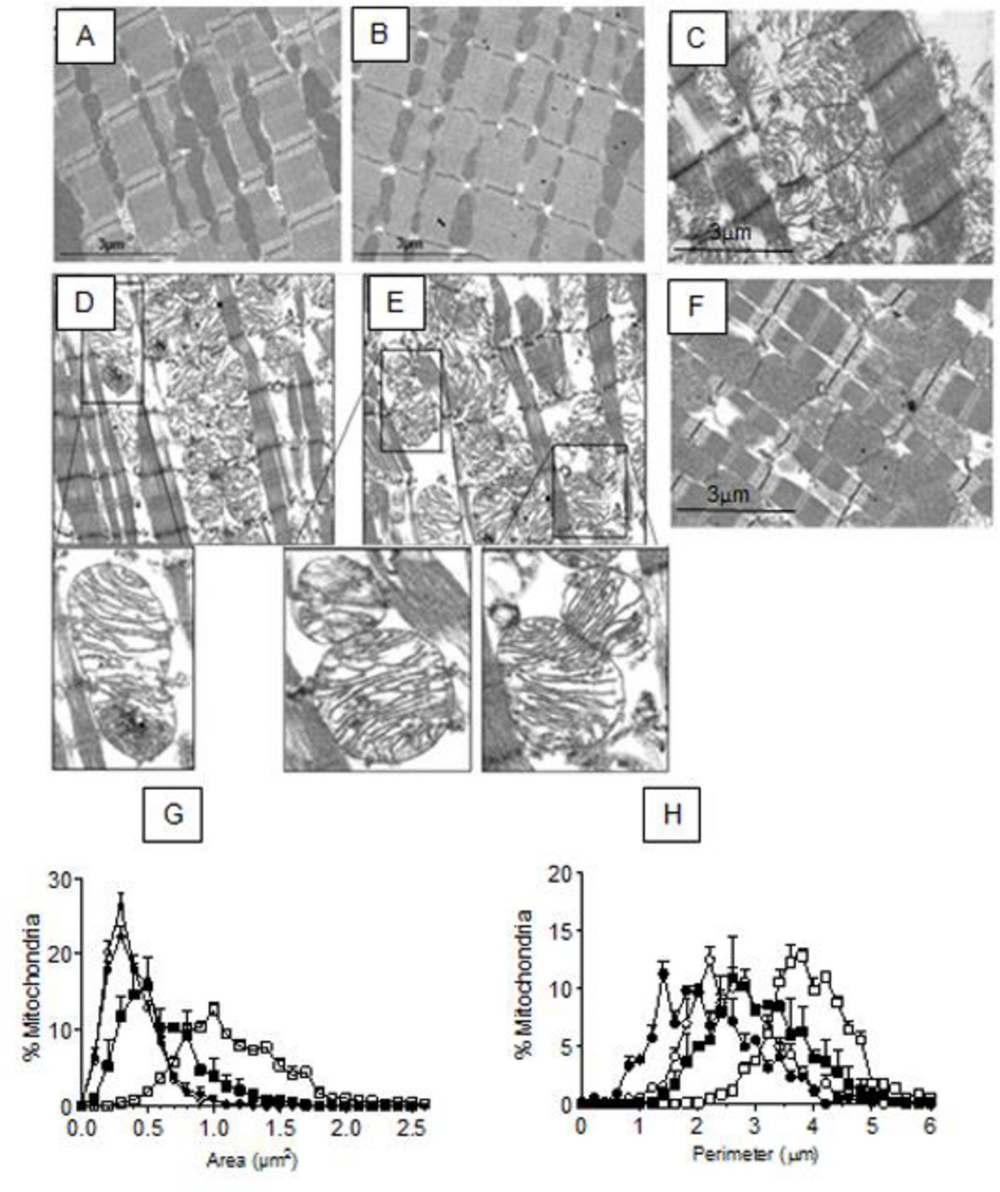
Effect of ixazomib on mitochondrial morphology in ischemic hearts. Transmission electron microscope images of (A) a control heart, (B) a control heart perfused with IXA, (C) a heart subjected to ischemia. (D and E) are images of an ischemic heart captured with a high-resolution scanning electron microscopy. Insets correspond to amplifications of the indicated zones in (D) and (E) showing mitochondrial cristae aberrations such as transversal discontinuity. (F) a heart subjected to ischemia in the presence of IXA (G) Frequency distribution of mitochondrial area and (H) perimeter of mitochondria in each experimental group. Symbols: C, white circles; C+IXA, black circles; I, white squares; I + IXA, black squares. Data on (G) and (H) are the average of 600–800 mitochondria.

The results shown above suggest that IXA protects the mitochondria during ischemia. To test this observation at a morphological level, we analyzed transmission electron microscopy images of cardiac tissue subjected to ischemia in the presence or absence of the drug. A longitudinal section of a control heart not subjected to ischemia, in which interfibrillar mitochondria arranged between sarcomeres can be appreciated, is shown in Fig 3A. IXA did not alter the mitochondrial ultrastructure under control conditions (Fig 3B). Fig 3C shows a section of cardiac tissue after 30 minutes of global ischemia. Ischemia provoked significant tissue damage, as demonstrated by disorganized sarcomeres and swollen mitochondria with disorganized cristae and a less electron-dense matrix (Fig 3C). Higher resolution images of particularly swollen mitochondria (increased area and lower electron density) accompanied by aberrant cristae in terms of transversal continuity and frequency are shown in Fig 3D and 3E. These changes were markedly reduced by the presence of IXA during ischemia (Fig 3F) indicating that proteasome inhibition protected the mitochondrial structure. Average mitochondrial morphological parameters after ischemia in the presence and absence of IXA are shown in Table 1. In summary, ischemia increased mitochondrial area (Fig 3G, Table 1) and perimeter (Fig 3H, Table 1) due to swelling and both indices were significantly decreased when ischemia was performed in the presence of IXA.

**Table 1:** Mitochondrial morphological parameters.

	Control	Ischemia	Control + IXA	Ischemia + IXA
Area (μm^2^)	0.374±0.004	1.161±0.034*	0.396±0.025	0.602±0.106^#^
Perimeter (μm)	2.648±0.081	3.938±0.058*	2.753±0.092	3.076±0.287^#^
Circularity	0.664±0.028	0.912±0.005*	0.658±0.019	0.743±0.046^#^

The values correspond to the averages ± the standard deviation. 600–800 mitochondria were counted in three different hearts of each experimental group.

*p<0.05 vs Control

# p<0.05 vs ischemia, Kruskal-Wallis test followed by Dunn`s test.

### Inhibition of proteasome CT-like activity attenuates the increase in left ventricular end diastolic pressure and reduces infarct size at the end of reperfusion

**Fig 4:**
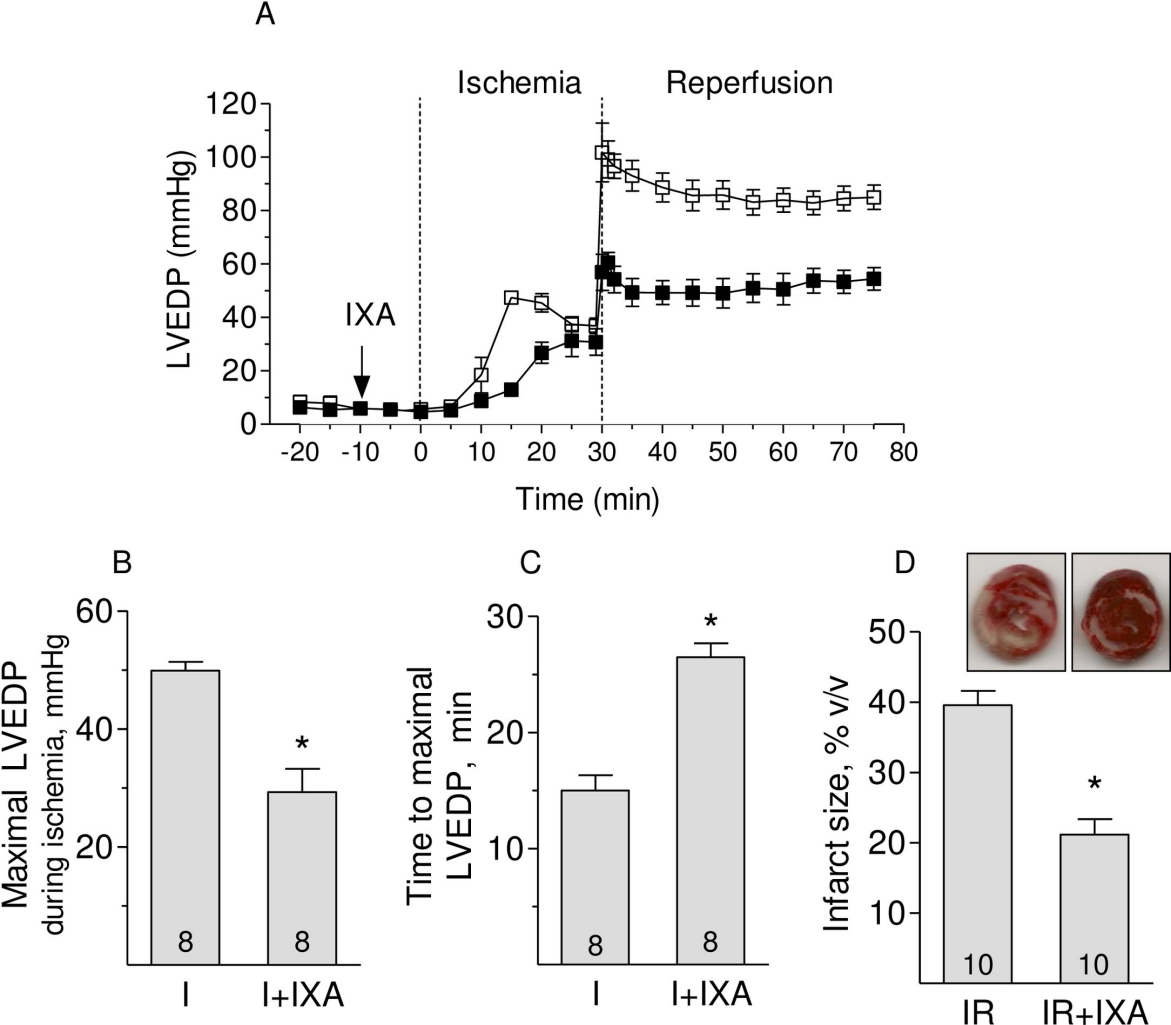
Effect of Ixazomib on left ventricular end-diastolic pressure and on the infarct size. (A) Left ventricular end-diastolic pressure (LVEDP) in isolated hearts during global ischemia and reperfusion in the presence (black squares) or absence (white squares) of IXA. (B) Maximal LVEDP and (C) time to maximal LVEDP during ischemia in the presence or absence of IXA were determined from records similar to the examples shown in (A) (D) Infarct size in the presence or absence of IXA. Number of hearts is shown in each bar. *p<0.05 Student’s t test.

Isolated hearts subjected to global ischemia stop beating in a few minutes and experience a progressive increase in the left ventricular end-diastolic pressure (LVEDP) starting at around 10 minutes of ischemia (Fig 4A). Reperfusion strongly exacerbated the increase in LVEDP that remained elevated during the following hour of reperfusion (Fig 4A). In the presence of IXA, the increase in LVEDP during ischemia was smaller and developed later, indicating that the myocardium remained viable for a longer period (Fig 4A). Likewise, LVEDP after one hour of reperfusion was lower in hearts treated with IXA (Fig 4A).These effects are summarized in Fig 4B and 4C. The reductions in these variables imply a decrease in tissue damage during ischemia, resulting in a significantly lower infarct size in the presence of IXA (Fig 4D).

## Discussion

This work extends our prior findings on the protective effects of IXA during myocardial ischemia [6]. The previously-observed decrease in infarct size in the presence of IXA prompted us to study the effect of this inhibitor on mitochondrial morphology and function during ischemia. Due to its elevated energy requirements, the heart is highly dependent on mitochondrial respiration, and practically all of the ATP required by the heart is generated by oxidative phosphorylation. Interruption of the blood supply causes a rapid drop in ATP production, with a consequent decline in contractile force and loss of cellular homeostasis. Oxygen deprivation provokes time-dependent mitochondrial damage that eventually becomes irreversible and produces cell death. Therefore, mitochondria are important targets for cardioprotection. We specifically analyzed the effect of IXA on Mfn2 as this protein plays a critical role not only in mitochondrial dynamics, but also in communication between the mitochondria and sarcoplasmic reticulum [9], as well as in other aspects of cell metabolism [20]. IXA was very effective in reducing mitochondrial damage during ischemia, as indicated by the preservation of mitochondrial morphology and improvement in mitochondrial function indices such as oxygen consumption, membrane potential, and glutathione content. Importantly, IXA blocked the release of cyt c from the mitochondria into the cytosol, which otherwise would initiate activation of the intrinsic apoptosis pathway [21], contributing to tissue death. The loss of cyt c would also contribute to the decrease in ATP generation during ischemia, even before oxygen is depleted. Moreover, cytc is a ROS scavenger. Loss of cyt c, therefore, would further contribute to mitochondrial damage, as suggested by the decrease in mitochondrial GSH levels. Consistently, IXA delayed the onset and decreased the amplitude of LVEDP, resulting in a significant reduction in infarct size.

The role of Mfn2 in mitochondrial structure and function in adult hearts is complex and not yet fully understood. IXA not only prevented the degradation of Mfn2 during ischemia but also, as we showed before, of RyR2 [6]. Since both proteins are present in SR-mitochondria associated membranes, these results suggest that the preservation of this interaction is important for cell survival under stress. In addition to the above-mentioned involvement in mitochondrial metabolism and SR-mitochondria communication, Mfn2 also protects neurons from apoptotic death following ischemia/reperfusion by modulating mitophagy, a process needed to rid the cells of damaged mitochondria [22]. Certainly, Mfn2 is unlikely to be the only protein preserved by proteasome inhibition during ischemia, but our data suggests that avoiding its degradation has an important role in the protection conferred by IXA.

The first attempts to inhibit the proteasome as a therapeutic measure against ischemia/reperfusion injury were highly controversial. Beneficial [23,24] or deleterious [25,26] effects were observed in various models of ischemia using different inhibitors and concentrations. We [6] and others [27,28] have shown that the degree of proteasome inhibition is critical to the protective effect. As shown in our previous work, the IXA concentrations used here only inhibit CT-like activity, without affecting proteasomal activities of caspase or trypsin-like activity. Inhibition of caspase-like activity associated with higher concentrations of IXA may cause undesired effects [6].

Most studies on ischemia/reperfusion injury focus on the injury produced at reperfusion, since ischemia typically occurs without prior warning. Nevertheless, irreversible damage and cell death begin during ischemia, and our results emphasize the need to protect the mitochondria during the ischemic period itself. Pharmacological approaches to prevent mitochondrial damage have yet to be translated into clinically-relevant therapeutic measures against myocardial ischemia. One of the reasons for this lack of success may be that many of the relevant experimental drugs are not currently approved for human use. Proteasome inhibition by IXA may be a feasible way to protect the heart from ischemic damage in cases where it is known in advance that ischemia will occur (cardiac surgery with extracorporeal circulation, for instance), as the drug is currently used in patients with multiple myeloma [29]. Furthermore, the IXA concentrations used in this study are similar to plasma concentrations of IXA in multiple myeloma patients [30], suggesting that a significant protective effect against myocardial ischemia can be achieved with pharmacological concentrations of the drug.

### Limitation of the study

We acknowledge the following limitations that should be addressed in future studies: a) ROS formation was not directly assessed during ischemia. This determination is challenging in Langendorff perfused hearts and it was not possible to perform under our protocol of ischemia. B) It would have been also desirable to determine the extent of mitochondrial fusion and fission in hearts treated with IXA. However, there are methodological restrictions to effectively measure mitochondrial fusion or fission in the heart. c) The effect of IXA on changes in gene expression (mRNA levels) associated with cardiac energetics was not investigated in the present work. It would be important to determine changes in mRNA levels associated with cardiac energetics within the first 30 min as previously reported in other studies [31, 32].

## Conclusion

Given its low toxicity and adequate tolerance [33], ixazomib may be a useful drug to protect cardiac mitochondria in cases of programmed ischemia.

## Supporting information

S1 Fig Critical mitochondrial proteins remained unchanged after 30 minutes of ischemia in isolated rat hearts. Proteins were quantified by Western blots in whole heart homogenates from control or ischemic hearts. The antibodies used were the following: anti Mitofusin 1 (cat # ab104274, Abcam, Cambridge, UK; anti FIS-1 (cat # PA5-22142, Thermo Scientific, Waltham, MA, USA); anti OPA-1 (cat # sc-367890, Santa Cruz Biotechnology, Dallas, TX, USA); COX IV (cat # 4844, Cell Signaling Technologies, Danvers, MA, USA); Cyclophilin D (cat # AP1035, Merck, Burlington, MA, USA). Protein content was normalized by the content of GAPDH (cat # G9547, Sigma Chemicals Co., St. Louis, MO). Bars show Mean ± S.E.M values of western blots like those shown on top, obtained in 4 different hearts.
(TIF)
