# Supplementary figures and images for: Inhibition of chymotrypsin-like activity of the proteasome by ixazomib prevents mitochondrial dysfunction during myocardial ischemia
Source: PLoS One. 2020 May 26;15(5):e0233591. doi: 10.1371/journal.pone.0233591 (PMC7250417; doi:10.1371/journal.pone.0233591)

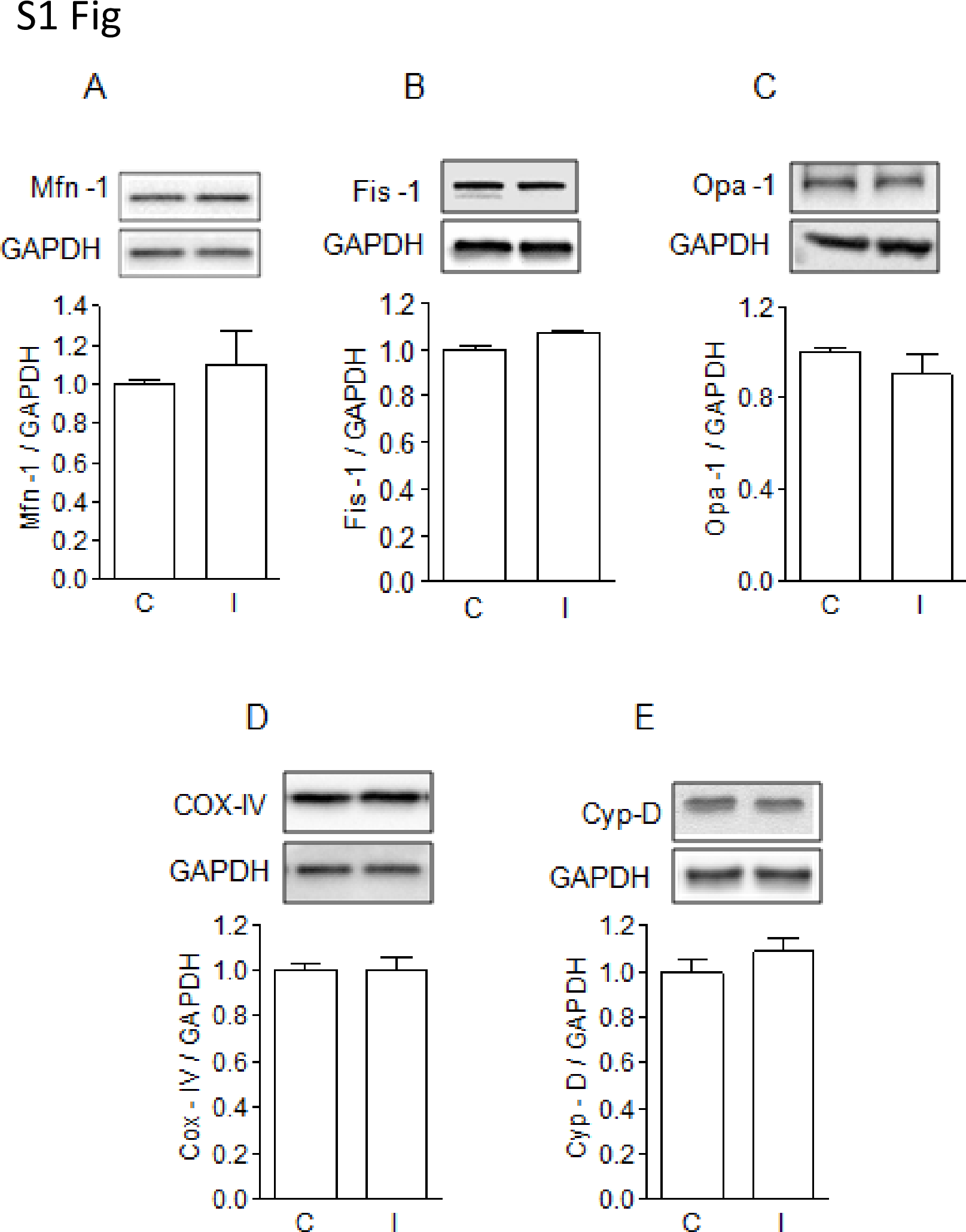

Supplement: S1 Fig — Proteins were quantified by Western blots in whole heart homogenates from control or ischemic hearts. The antibodies used were the following: anti Mitofusin 1 (cat # ab104274, Abcam, Cambridge, UK; anti FIS-1 (cat # PA5-22142, Thermo Scientific, Waltham, MA, USA); anti OPA-1 (cat # sc-367890, Santa Cruz Biotechnology, Dallas, TX, USA); COX IV (cat # 4844, Cell Signaling Technologies, Danvers, MA, USA); Cyclophilin D (cat # AP1035, Merck, Burlington, MA, USA). Protein content was normalized by the content of GAPDH (cat # G9547, Sigma Chemicals Co., St. Louis, MO). Bars show Mean ± S.E.M values of western blots like those shown on top, obtained in 4 different hearts. (TIF) [file pone.0233591.s001.tif]
